# Endovascular treatment of lower extremity arteries is associated with an improved outcome in diabetic patients affected by intermittent claudication

**DOI:** 10.1186/1471-2482-12-S1-S19

**Published:** 2012-11-15

**Authors:** Giuseppe Giugliano, Cinzia Perrino, Vittorio Schiano, Linda Brevetti, Anna Sannino, Gabriele Giacomo Schiattarella, Giuseppe Gargiulo, Federica Serino, Marco Ferrone, Fernando Scudiero, Andreina Carbone, Antonio Bruno, Bruno Amato, Bruno Trimarco, Giovanni Esposito

**Affiliations:** 1 Department of Clinical Medicine and Cardiovascular and Immunological Sciences, “Federico II” University”, via Pansini 5, 80131, Naples, Italy; 2 Department of General, Geriatric, Oncologic Surgery and Advanced Technologies, “Federico II” University”, via Pansini 5, 80131, Naples, Italy

## Abstract

**Background:**

Lower extremity peripheral arterial disease (LE-PAD) is a highly prevalent condition among diabetic patients, associated with reduced walking capacity and a high incidence of cardiovascular events. Endovascular revascularization of lower extremities arteries improves walking performance and quality of life of diabetic patients affected by intermittent claudication, but few studies evaluated the impact of revascularization on cardiovascular outcome in this high-risk population. Accordingly, in the present study we evaluated if leg-ischemia resolution by effective lower limbs percutaneous revascularization can also impact cardiovascular outcome in a homogeneous group of diabetic patients affected by intermittent claudication.

**Methods:**

236 diabetic patients affected by LE-PAD at stage II of Fontaine’s classification, with ankle/brachial index ≤0.90 and one or more hemodynamically significant stenosis in at least one artery of the ileo-femoro-popliteal axis were enrolled in the study. According to the Trans-Atlantic Inter Society Consensus II recommendations, 123 (52.1%) underwent percutaneous transluminal angioplasty (PTA group), while 113 (47.9%) underwent conservative medical therapy only (MT group). The incidence of major cardiovascular events (cardiovascular death, myocardial infarction, ischemic stroke, coronary or carotid revascularization) was prospectively analyzed with Kaplan-Meier curves and the risk of developing a cardiovascular event calculated by Cox analyses.

**Results:**

No baseline difference in cardiovascular risk factors were observed between the PTA and MT groups, except for a lower prevalence of males in PTA group (74.8% vs. 85.8%, p=0.034). Furthermore, patients in the PTA group showed a worse walking capacity as expressed by maximum walking distance (108.7 ± 300.9 vs 378.4 ± 552.3 meters, p<0.001). During a median follow-up of 20 months (12.0-29.0), the incidence of cardiovascular events was markedly lower in patients in the PTA group with respect to patients in the MT group (7.3% vs. 22.1%, p=0.001), and patients of the MT group had at Cox analysis a 3.9 increased risk with respect to PTA group, after adjustment for potential confounding factors (95% CI 1.1-15.3, p=0.049).

**Conclusions:**

The present study shows that lower limbs revascularization of diabetic patients affected by intermittent claudication, in addition to improve walking performance, is associated with a reduction in the incidence of future major cardiovascular events.

## Background

Cardiovascular disease (CVD) represents the leading cause of death in western countries affecting especially middle-age people [1-3]. One of the most important risk factor, widely recognized as an independent predictor of outcome, is diabetes [4,5]. Diabetes affects nearly all vascular beds and in affected patients the risk of morbidity is about twice that of age-matched non diabetic patients [6]. The metabolic disorders accompanying diabetes seem to accelerate the progression of atherosclerosis and indeed more than half of diabetics will die as a result of a cardiovascular ischemic event [7,8].

Lower extremity peripheral arterial disease (LE-PAD), one of the main expressions of atherosclerosis, is a highly prevalent pathological condition among diabetic patients associated with reduced walking capacity and a high incidence of developing future cardiovascular ischemic events [9-14]. When LE-PAD develops in the setting of diabetes, it portends a significantly increased danger to both life and limb function [15,16].

While revascularization should be attempted without delay in all patients presenting with critical limb ischemia, whenever technically possible, the management of intermittent claudication varies depending on the severity of walking impairment and the associated impact of this functional disability on individual lifestyle [17]. According to the most recent guidelines, LE-PAD patients with limited walking capacity should be managed with limb revascularization procedures only when exercise and/or drug therapy fail to improve symptoms [17-23]. Both open repair/bypass surgery or percutaneous trans-luminal angioplasty (PTA) are effective revascularization approaches, and the choice is based upon the number, length and localization of the stenosis/occlusion, surgery risk score and patient preference [3,18]. PTA of the lower limbs is effective in improving not only functional status and quality of life in claudicants, [21,23] but it is also associated with improved cardiovascular outcome [9]. Whether these effects can be also observed in patients with diabetes and intermittent claudication is currently unknown. Thus, we conducted a prospective study to evaluate whether effective endovascular revascularization by PTA might be associated with a reduction in cardiovascular events compared to medical therapy only in a homogeneous cohort of diabetic patients affected by LE-PAD and intermittent claudication.

## MethodsE

### Study population

Consecutive diabetic patients referred to our vascular laboratory for suspected intermittent claudication were screened for enrollment in this study. Criteria for study entry were all of the following: 1) diagnosis of diabetes mellitus; 2) LE-PAD at stage II of Fontaine’s classification (intermittent claudication); 2) ABI ≤0.90; 3) one or more hemodynamically significant stenosis in at least one artery of the ileo-femoro-popliteal axis at B-mode ultrasound. Exclusion criteria were: 1) critical limb ischemia; 2) previous lower limb revascularization; 3) recent acute coronary or cerebrovascular ischemic events (6 months); 4) recent coronary or carotid revascularization procedures (6 months); 5) abnormal myocardial ischemia stress test at enrollment; 6) de-compensated heart failure; 7) malignant neoplasia or significant hepatic, renal, or inflammatory disease.

According to the inclusion/exclusion criteria, 252 consecutive diabetic patients affected by intermittent claudication were selected. All patients were treated with maximal medical therapy and encouraged to engage regular physical exercise for at least three months. After this time, patients complaining a severe disability caused by claudication, unable to perform normal work or with very serious impairment of daily life activities despite maximal medical therapy and regular physical exercise (n=139) were selected for angiography and eventually revascularization, while the remaining 113 patients were managed with medical therapy only (MT group). Among the patients initially selected for revascularization, 2 patients refused to undergo angiography, and were excluded from the study. Based on the angiograms, 12 patients displaying TASC D lesions were excluded from the study, while 125 underwent endovascular revascularization. Following PTA, only 123 patients displayed a successful angiographic result (2 patients showed a residual stenosis >30%), and therefore were included in the study (PTA group). All participants gave written informed consent to the study, which was approved by our institutional ethics committee.

### Clinical assessment

In each patient, clinical history and risk factors were assessed at first visit. Smokers included current and former smokers. Hypertension was diagnosed if systolic arterial pressure exceeded 140 mmHg and/or diastolic arterial pressure exceeded 90 mmHg on repeated measurements, or if the patient used antihypertensive drugs. Hypercholesterolemia was diagnosed if plasma total cholesterol exceeded 200 mg/dl, plasma low-density lipoprotein cholesterol exceeded 130 mg/dl, or if the patient used lipid-lowering drugs because of a history of hypercholesterolemia. Hospital records documented previous cardiovascular events.

### ABI and maximum walking distance assessment

ABI was measured at the first visit after participants had rested supine for 5 minutes. The systolic blood pressure in both brachial arteries and the ankle systolic blood pressure for the right and left posterior tibial and dorsalis pedis arteries were measured using a Doppler probe. The ABI for each leg was then determined using the higher of the two readings from either the posterior tibial or dorsalis pedis arteries, and the higher of the two brachial readings. The lower ABI of the two legs was used for diagnostic purposes and as predictor of future cardiovascular events. Maximum walking distance (MWD) was tested by treadmill (speed 3 km/h, inclination 10%) at the first visit.

### Endovascular procedure

Percutaneous Transluminal Angioplasty (PTA) was performed after diagnostic angiography and intra-venous injection of 70 U/kg of unfractionated heparin. Bailout nitinol self-expanding stent implantation was performed when a suboptimal angiographic result was obtained. Successful angioplasty was defined by a final angiogram with residual stenosis <30%.

### Assessment of cardiovascular events

Patients underwent regular follow-up clinical examinations at our Institution at 3-month intervals. The occurrence of cardiovascular death, myocardial infarction, ischemic stroke and coronary or carotid revascularizations was prospectively assessed. Cardiovascular deaths comprised fatal myocardial infarction, fatal stroke, sudden death, and death secondary to arrhythmia or refractory heart failure. The minimum follow-up period was 6 months. Medical records and death certificates of all patients who had an event were obtained and validated by a physician unaware of patient’s peripheral treatment. For patients who had more than 1 event, only the first was considered in the analysis.

### Statistical analysis

Statistical analyses were performed using SPSS 16.0 (SPSS, Inc., Chicago, IL, USA). Variables were expressed as absolute numbers and percentage or mean ± SD, with the exception of leukocyte count that was expressed as median and inter-quartile range because of its skewed distribution. Comparisons were made by t-test for unpaired samples, χ^2^ test, or Mann-Whitney U test, as appropriate. Cumulative event rates in the PTA vs. MT group were estimated by Kaplan-Meier curves and probability values by log-rank test.

Cox proportional hazard analyses were performed to verify if endovascular treatment was associated with a lower incidence of future cardiovascular events. The following covariates, known to be potential contributors of cardiovascular risk, were included in the adjusted model: age, sex, smoking, hypercholesterolemia, hypertension, baseline ABI, baseline maximum walking distance, and leukocyte count.

All statistical tests were two-sided. For all tests, a p-value <0.05 was considered statistically significant.

## Results

### Patients’ characteristics

Table 1 reports the baseline characteristics of the patients in the PTA and MT groups. There were fewer males in the PTA group with respect to MT group (74.8% vs. 85.8%, p=0.034) and, not surprisingly, the MT group was characterized by a better functional capacity (maximum walking distance: 378.4 ± 552.3 vs. 108.7 ± 300.9 meters, p<0.001). Conversely, no difference between the two groups was observed with respect to the prevalence of classic cardiovascular risk factors, cardiovascular co-morbidity, and baseline ABI.

**Table 1:** Baseline characteristics of the study population

	PTA Group	MT Group	p
	(n = 123)	(n = 113)	
Age (yr)	64.7 ± 9.7	66.2 ± 8.9	0.323
Males	92 (74.8)	97 (85.8)	0.034
***Risk factors***			
Hypercholesterolemia	98 (79.6)	79 (69.9)	0.152
Hypertension	112 (91.0)	183 (92.0)	0.757
Smoking	107 (87.0)	91 (80.5)	0.239
BMI	27.6 ± 5.3	26.5 ± 5.2	0.831
***LE-PAD severity***			
ABI	0.66 ± 0.18	0.65 ± 0.17	0.580
MWD (meters)	108.7 ± 300.9	378.4 ± 552.3	0.001
***Comorbidity***			
Previous MI	32 (26.0)	33 (29.2)	0.584
Previous stroke	3 (2.4)	1 (0.9)	0.360
***Inflammatory status***			
Leukocyte count (x 10^9^/L)	7.9 [6.6 – 9.3]	7.4 [5.9 – 9.1]	0.150

Values are n (%) or mean ± SD or median [interquartile range].

PTA = percutaneous transluminal angioplasty; MT = medical therapy; BMI = body mass index; LE-PAD = lower extremity peripheral arterial disease; ABI = ankle/brachial index; MWD = maximum walking distance; MI = myocardial infarction.

### Endovascular treatment and outcome

**Figure 1:**
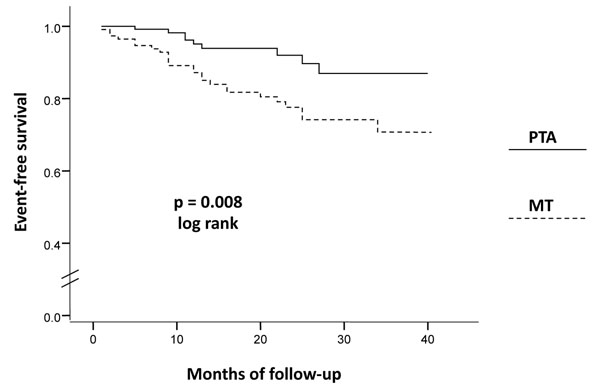


During a median follow-up of 20.0 months (interquartile range 12.0 – 29.0), 34 of the 236 patients (14.4%) had a major cardiovascular event, of which 25 (22.1%) occurred in the MT group, while only 9 (7.3%) occurred in the PTA group (p<0.001). Importantly, the PTA group was characterized by a lower rate of cardiovascular deaths, especially driven by a reduction in the rate of fatal MI (data not shown). Consistent with these results, Kaplan-Meier curves depicting the incidence during follow-up of total cardiovascular events showed a significant advantage in the PTA group vs. MT group (Figure 1).

At Cox analysis, patients in the MT group had a 2.68-fold increased risk (95% CI 1.24-5.74, p = 0.011) of developing a cardiovascular event with respect to patients in the PTA group. Notably, this association remained statistically significant after adjustment for the potential confounders indicated above (adjusted HR= 3.92, 95% CI 1.10-15.30, p = 0.049).

## Discussion

The present study demonstrates that successful revascularization of lower extremity arteries in diabetic patients affected by intermittent claudication is associated with a reduction in the incidence of major cardiovascular events. Notably, this association remains unaltered after accounting for possible confounders such as classic cardiovascular risk factors, previous myocardial infarction or stroke, maximum walking distance, leukocyte count and ABI, to date the most powerful prognostic indicator in LE-PAD [24,25]. These results might have important clinical implications, and might open a new scenario for diabetic patients affected by intermittent claudication, in which the indications to perform lower-extremity revascularization might be extended to the improvement of global cardiovascular outcome. At this regard, it is also important to emphasize that PTA of the lower limbs is now a safe and largely effective procedure [18,26].

Although patients undergoing PTA were characterized by worse baseline functional capacity compared to the MT group (Table 1), we observed a better cardiovascular outcome in patients undergoing lower extremity reperfusion strategy. Different mechanisms might be accounted for these beneficial effects associated to the revascularization of the ischemic leg. Firstly, PTA increases walking capacity [20,22], and such improvement in functional status induced by PTA might be responsible, at least in part, of the reduction of cardiovascular risk observed in our patients. Indeed, patients affected by intermittent claudication may be severely limited in their occupational and leisure-time physical activity [27,28] and a sedentary lifestyle is a risk factor for adverse cardiovascular events [29-31]. Another possible mechanism for the favourable effects observed in the PTA group might be inherent to the increase of ABI. Indeed, successful revascularization of lower extremities has been shown to improve the ABI, which is the most powerful prognostic indicator in LE-PAD patients [24,25]. Furthermore, PTA of the lower limbs has been also associated with the improvement of endothelial function [32], which plays an important role in the pathophysiology and natural history of lower extremities atherosclerotic disease [33], and may reduce the ischemia–reperfusion injury which promotes systemic inflammation [34]. At this regard, it is important to emphasize that an increased inflammatory status has been associated to the development and subsequent worsening of atherosclerosis including thrombotic complications, and that the elevation in circulating inflammatory markers increases the risk if ischemic cardiovascular events in LE-PAD [13].

## Conclusions

In conclusion, the present study provides evidence that effective lower limb revascularization by PTA in diabetic patients affected by intermittent claudication not only ameliorates functional status and alleviates symptoms, but is also associated with an improvement of cardiovascular outcome. Further studies are needed to understand the possible mechanisms underlying this result.

## List of abbrevations

CVD: Cardiovascular Disease; LE-PAD: Lower Extremity Peripheral Arterial Disease; PTA: percutaneous trans-luminal angioplasty; TASC: Trans-Atlantic Inter-Society Consensus; ABI: Ankle/Brachial Index.

## Competing interests

The authors declare that they have no competing interests.

## Authors’ contributions

GG, CP, VS, LB, AS, GGS, GG, FS, MF, FS, AC, AB: conception and design, interpetration of data, given final approval of the version to be published; BA, BT: critical revision, interpretation of data, given final approval of the version to be published; GE: conception and design, critical revision, given final approval of the version to be published.
